# Clinicopathological characteristics and mutational profile of KRAS and NRAS in Tunisian patients with sporadic colorectal cancer

**DOI:** 10.3906/sag-2003-42

**Published:** 2021-02-26

**Authors:** Donia OUNISSI, Marwa WESLATI, Rahma BOUGHRIBA, Meriam HAZGUI, Saadia BOURAOUI

**Affiliations:** 1 Laboratory of Colorectal Cancer Research UR12SP14, Mongi Slim Hospital, La Marsa Tunisia; 2 Faculty of Sciences of Tunis, University of Tunis El Manar, Tunis Tunisia; 3 Department of Pathology and Cytology, Mongi Slim Hospital, La Marsa Tunisia

**Keywords:** Sporadic colorectal cancer, *KRAS*, *NRAS*, real-time PCR, ARMS-PCR, Tunisia

## Abstract

**Background/aim:**

Colorectal cancer (CRC) is a major public health problem worldwide and in Tunisia due to its increasing rate of incidence. *KRAS* and *NRAS* mutations have become a pivotal part of CRC diagnosis, given their association to treatment resistance with antiepidermal growth factor receptor (EGFR) monoclonal antibodies. In this study, we aimed to screen for mutations in *KRAS* and *NRAS* genes in Tunisian patients with CRC and explore their correlations with clinicopathological features.

**Materials and methods:**

AmoyDx *KRAS* and *NRAS* mutation real-time PCR kits were used to screen for mutations in *KRAS* (exon 2) and *NRAS* (exons 2, 3, and 4) in 96 CRC tumors.

**Results:**

*KRAS* exon 2 mutations were found in 41.7% (40/96) of the patients. Codon 12’s most abundant mutations were G12D and G12V, followed by G12A, while G13D is the predominant mutation in codon 13. *KRAS* exon 2 mutations were associated with older patients (P = 0.029), left-sided tumors (P = 0.037), and greater differentiation (P = 0.044). The prevalence rate of *NRAS* mutations was 7.3%, mostly in exon 2. These mutations were associated with early stages of the disease (P = 0.039) and the absence of lymph node metastasis (P = 0.045).

**Conclusion:**

It can be inferred from this study that Tunisian CRC patients have a similar frequency of *KRAS* and *NRAS* mutations compared to those observed in other populations. Consequently, screening for *KRAS* and *NRAS* mutations is crucial for the orientation of therapies and the selection of appropriate candidates, while also helping to avoid unnecessary toxicity and increased costs for patients.

## 1. Introduction

Due to its increasing incidence and mortality rates, colorectal cancer (CRC) is a major global health concern [1]. This neoplasm ranks as the 3rd most common cancer among both men and women, with nearly 1.8 million new diagnosed cases each year [1,2].

The 2018 Global Cancer Statistics ranked CRC as the 2nd leading cause of cancer-related morbidity, with an estimated 881,000 cancer deaths worldwide [1].

As a public health problem worldwide and in Tunisia, CRC incidence has increased over the past 20 years [3,4]. In Tunisia, colorectal carcinoma is considered to be the most frequent digestive cancer [5,6].

The adenoma-carcinoma sequence is a multistep process that starts with genetic alterations in early adenoma, and accumulation transforms it into carcinoma [7]. 

Studies pinpointed 3 major pathways that are responsible for genomic instability in CRC: chromosomal instability, microsatellite instability, and CpG island methylator phenotype [8]. Most colorectal cancers arise through the chromosomal instability pathway due to the accumulation of somatic mutations in protooncogene ( *KRAS* ) and tumor suppressor genes such as *APC* and *TP53* [8].

*KRAS* mutations are considered to be an early event in tumori­genesis [8–11]. In colorectal cancer, approximately 30% to 50% of tumors harbor these mutations [11–13]. Approximately 90% of *KRAS* mutations are located in codons 12 and 13 [11,14]. They are mostly single-nucleotide point mutations, particularly G>A transitions and G>T transversions [15,16].

As a main effector molecule in the epidermal growth factor receptor (EGFR) signaling pathway, mutant *KRAS* tumors exhibit resistance to EGFR-targeted therapies [17]. Subsequently, the American Society for Clinical Oncology (ASCO) and the National Comprehensive Cancer Network (NCCN) have recommended that *KRAS* gene mutation analysis occur before anti-EGFR therapies [18,19].

In 2009, the US Food and Drug Administration (FDA) and European Medicines Agency (EMEA) deemed the 2 EGFR antagonists, cetuximab and panitumumab, as “not recommended” for the treatment of patients with metastatic CRC (mCRC) harboring *KRAS* mutations [16,20].

However, most patients with *KRAS* codons 12/13 wild-type colorectal cancer still fail to respond to anti-EGFR therapy, suggesting the involvement of other mutations [21,22].

In this context, *NRAS* , a member of the *RAS* family, is found to be mutated in 1%–7% of colorectal cancers [23]. In fact, recent research showcased that mutations in *KRAS* exons 3 and 4 and *NRAS* gene exons 2, 3, and 4 are associated with resistance to the anti-EGFR antibody or to poor prognosis in mCRC [24,25]. Thus, EMEA and ASCO, have made it mandatory to investigate exons 2, 3, and 4 of both *KRAS* and *NRAS* prior to the use of any novel targeted therapies such as anti-EGFR treatments [2,26].

In conclusion, the *RAS* gene family ( *KRAS* and *NRAS* ) status allows for the better the orientation of therapies and, therefore, make it possible for patients to avoid unnecessary toxicity and additional costs related to care [8,16,22].

In view of these points, our work aims to screen for mutations in *KRAS* and *NRAS* genes in Tunisian patients with sporadic colorectal cancer and explore their correlations with clinicopathological features. 

## 2. Materials and methods

### 2.1. Patients and tumor samples

We conducted this retrospective study from 2010 to 2018 with the information from the cases of 96 sporadic CRC patients. The study protocol was approved by the Ethics Committee of Mongi Slim Hospital, La Marsa, Tunisia.

Mutational analyses were performed on frozen specimens taken from patients who underwent colorectal tumor resection at the Department of Surgery on archival paraffin-embedded tissue blocks, either on primary or metastatic samples and preserved at the Department of Pathology and Cytology of the Mongi Slim Hospital, La Marsa, Tunisia. 

Clinicopathological features (age, sex, tumor location, histological type, differentiation, depth of invasion, TNM (tumor node metastasis) stage, and lymph node metastasis were collected for each patient from surgical and pathological records.

### 2.2. Samples selection and DNA extraction

After evaluating standard hematoxylin/eosin-stained slides from primary and metastatic colorectal adenocarcinomas, appropriate samples were specifically selected by a pathologist to include predominately tumor cells without significant necrosis or inflammation. Five 5.0-μm-thick unstained sections were cut from the preselected paraffin blocks; for the frozen specimens, 25 mg was taken from each sample.

DNA was extracted using the PureLink Genomic DNA Mini Kit (Invitrogen, Carlsbad, CA, USA), following the manufacturer’s instructions. 

DNA concentration was assessed using a Qubit dsDNA HS (high sensitivity) Assay kit (ThermoFisher Scientific, Waltham, MA, USA) on a Qubit 2.0 Fluorometer (ThermoFisher Scientific), according to manufacturer’s instructions.

### 2.3. Analysis of KRAS and NRAS gene mutations by amplification-refractory mutation system-polymerase chain reaction (ARMS-PCR)

The AmoyDx *KRAS* Seven Mutations Detection and *NRAS* Mutations Detection kits (Amoy Diagnostics Co., Xiamen, China) were used to detect the *KRAS* and *NRAS* status of each DNA sample. Both kits are Chinese Food and Drug Administration (CFDA) approved for clinical use in China and marked for in vitro diagnostic (IVD) use in Europe by the Conformité Européenne (CE) 

These highly sensitive kits are based on a patented technology ADxARMS, enabling the detection of 1% mutant DNA in a background of 99% normal DNA in a 10-ng DNA sample, while ensuring minimal false negatives.

The AmoyDx *KRAS* Seven Mutations Detection Kit is designed to accurately identify the 7 most common activating *KRAS* mutations in codons 12 and 13 (Table 1).

**Table 1:** KRAS and NRAS Mutations detected with the AmoyDx kit.

Gene	Exon	Codon	Mutation	Base change	Cosmic ID
	2	12	G12C	34G>T	516
			G12S	34G>A	517
			G12R	34G>C	518
KRAS			G12V	35G>T	520
			G12D	35G>A	521
			G12A	35G>C	522
		13	G13D	38G>A	532
NRAS	2	12	G12C	34G>T	562
			G12S	34G>A	563
			G12D	35G>A	564
			G12A	35G>C	565
			G12V	35G>T	566
		13	G13R	37G>C	569
			G13D	38G>A	573
			G13V	38G>T	574
	3	59	A59D	176C>A	253327
		61	Q61K	181C>A	580
			Q61L	182A>T	583
			Q61R	182A>G	584
			Q61H	183A>C	586
	4	117	K117N	351G>C	_
			K117N	351G>T	_
		146	A146T	436G>A	27174

The AmoyDx *NRAS* Mutation Detection Kit is intended to meticulously detect 16 hotspot somatic mutations in codons 12, 13, 59, 61, 117, and 146 of the *NRAS* gene (Table 1).

The extracted DNA quality was evaluated by amplifying a housekeeping gene and using the HEX channel provided with the kit.

PCR reactions were performed using a Stratagene Mx3005P (Agilent Technologies, Inc., Santa Clara, CA, USA) under the following conditions: 5 min incubation at 95 °C, followed by 15 cycles of 95 °C for 25 s, 64 °C for 20 s, 72 °C for 20 s, and then 31 cycles of 93 °C for 25 s, 60 °C for 35 s, and 72 °C for 20 s.

The fluorescent signal was collected from the FAM and HEX channels. It is important to note that every PCR run must contain one PC (positive control) and one NTC (no template control). *KRAS* and *NRAS* mutation status was determined according to the Ct value as indicated in the manufacturer’s instructions.

### 2.4. Statistical analysis 

All statistical analyses were performed using SPSS software, version 20 (SPSS, Inc., Chicago, IL, USA). Associations between variables were tested with the chi-square (χ2) test. A probability (P) value of less than 0.05 was considered to be statistically significant.

## 3. Results

### 3.1. Patient and tumor characteristics

In our 96 CRC patients, CRC prevalence was higher in males (62.5%, 60/96) than in females (37.5%, 36/96). The mean age of the Tunisian patients, at tumor resection, was 62.4 years old, ranging from 23 to 92 years of age. 

Regarding the histological subtypes of our series, 69.8% (67/96) of tumors were nonmucinous (NMC), and 30.2% (29/96) were mucinous adenocarcinomas (MC). 

The tumors were graded according to the WHO criteria (World Human Organization Classification of Tumours of the Digestive System, 4th Edition) [27] as follows: 47 (49%) were well-differentiated, 46 (47.9%) were moderately differentiated, and 3 (3.1%) were poorly differentiated.

A total of 71 samples (73.96%) were located in the left colon and 25 (26.04%) in the right colon.

Histologic classification of tumors was made according to the international TNM staging system based on the 8th edition of the American Joint Committee on Cancer (AJCC; 8th edition) [28]. 

Specimens were taken from 70 primary tumors (72.92%) and 26 metastasis (27.08%), distributed between 32 cases in the primary stage (stages I and II) and 64 cases in the advanced stage (stages III and IV).

### 3.2. Distribution of KRAS and NRAS mutations in colorectal carcinomas

The distribution of *KRAS* and *NRAS* mutations in the 96 CRC patient samples is presented in Table 2.

**Table 2:** Distribution of KRAS and NRAS mutations in the 96 CRC patient samples.

Gene	Exon	Codon	Mutation	Numbers of mutations(% of 96)
KRAS	2	12,13	G12D, G12A, G12V, G12S, G12R, G12C, G13D	47 (48.96)
NRAS	2	12,13	G12D, G12S,G13D, G13R, G12C, G12V, G12A, G13V	5 (5.21)
NRAS	3	59,61	A59D, Q61R, Q61K, Q61L, Q61H	3 (3.12)
NRAS	4	117,146	K117N, A146T	0

*KRAS* exon 2 mutations were observed in 41.7% (40/96) of the cases, and 7 cases had 2 concomitant mutations. Therefore, in a total of 40 cases, we had 47 mutations in *KRAS* exon 2, distributed as follows: 40 (85%) were detected in codon 12, and 7 (15%) were identified in codon 13.

The most prevalent mutations were G12D and G12V at 25.5% (12/47) each, followed by G12A at 17% (8/47); then G13D at 14.9% (7/47), G12R and G12C at 6.4% (3/47) each, and G12S at 4.3% (2/47).

Mutations outside *KRAS* exon 2 were observed in the *NRAS* gene in 7.3% (7/96) of the cases. One case showed 2 *NRAS* mutations. These 8 mutations were distributed as follows: 62.5% (5/8) in exon 2 (codons 12 or 13); 37.5% (3/8) in exon 3 (codon 61); and none in exon 4 (codon 146).

Figure displays the different mutation profiles, shown in subfigures (a), (b), and (c).

**Figure:**
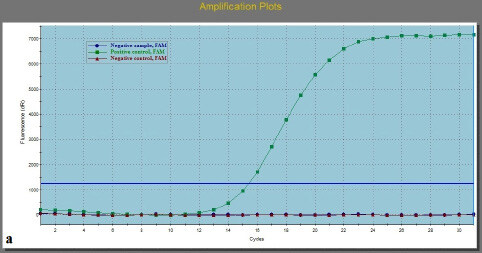

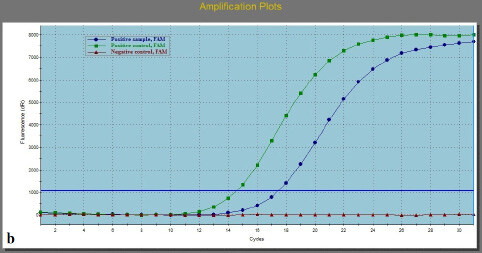

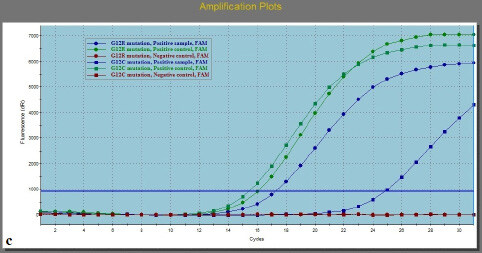
Amplification plots of: (a) wild-type sample; (b) sample with one mutation; (c) sample with 2 mutations.

In our study, none of the patients harbored a simultaneous mutation in *KRAS* (exon 2) and *NRAS* (exons 2, 3, and 4). Therefore, these mutations were mutually exclusive.

### 3.3. Association between KRAS/NRAS mutations and clinicopathological features

A summary of the relationships between *KRAS* and *NRAS* mutations and various clinicopathological features is provided in Table 3.

**Table 3:** Correlation between KRAS/ NRAS mutations and clinicopathological features.

Clinicopathological features	Number	KRAS status			NRAS status		
		Wild typeN = 56 (%)	Mutant typeN = 40 (%)	P-value	Wild typeN = 89 (%)	Mutant typeN = 7 (%)	P-value
Age (years)							
≥60 (n = 65)	65	33 (58.9)	32 (80)	0.029	62 (69.7)	3 (42.9)	0.149
<60 (n = 31)	31	23 (41.1)	8 (20)		27 (30.3)	4 (57.1)	
Sex							
Male (n = 60)	60	36 (64.3)	24 (60)	0.669	54 (60.7)	6 (85.7)	0.184
Female (n = 36)	36	20 (35.7)	16 (40)		35 (39.3)	1 (14.3)	
Tumor location							
Right colon (n = 25)	25	19 (33.9)	6 (15)	0.037	22 (24.7)	3 (42.9)	0.260
Left colon (n = 71)	71	37 (66.1)	34 (85)		67 (75.3)	4 (57.1)	
Histological type							
NMC (n = 67)	67	35 (62.5)	32 (80)	0.066	64 (71.9)	3 (42.9)	0.120
MC (n = 29)	29	21 (37.5)	8 (20)		25 (28.1)	4 (57.1)	
Differentiation							
Well (n = 47)	47	22 (39.3)	25 (62.5)		45 (50.6)	2 (28.6)	0.151
Moderate (n = 46)	46	31 (55.4)	15 (37.5)	0.044	42 (47.2)	4 (57.1)	
Poor (n = 3)	3	3 (5.3)	0 (0)		2 (2.2)	1 (14.3)	
Stages							
I, II (n = 32)	32	19 (33.9)	13 (32.5)	0.884	27 (30.3)	5 (71.4)	0.039
II, IV (n = 64)	64	37 (66.1)	27 (67.5)		62 (69.7)	2 (28.6)	
Lymph node metastasis							
No (n = 33)	33	21 (37.5)	12 (30)	0.446	28 (31.5)	5 (71.4)	0.045
Yes (n = 63)	63	35 (62.5)	28 (70)		61(68.5)	2 (28.6)	

*KRAS* mutations were much higher in older patients (>60 years old) (80% vs. 20%, P = 0.029) and were significantly more prevalent in the left side of the colon than on the right side (85% vs. 15%, respectively; P = 0.037). Meanwhile, these mutations were significantly associated with well-differentiated tumors but less with moderately and poorly differentiated tumors (62.5% vs. 37.5% vs. 0%, respectively; P = 0.044). 

Although *KRAS* mutation frequency is higher in NMC (80%), the difference is not statistically significant (P = 0.066).

There was no significant relationship between *KRAS* mutations and sex (P = 0.669), lymph node metastasis (P = 0.446), or tumor stage (P = 0.884).

*NRAS* mutations were more frequent in stages I and II (71.4%), compared with stage III and IV cancers (28, 6%) (P = 0.039) and were associated with the absence of lymph node metastasis N0 (P = 0.045).

However, no significant relationship was observed between *NRAS* mutations and sex (P = 0.184), age (P = 0.149), tumor location (P = 0.260), histological type (P = 0.120), or tumor differentiation (P = 0.151).

## 4. Discussion

As the 3rd most common cancer among men and women, CRC has increased in terms of incidence and mortality worldwide and in Tunisia [1,3]. The 2018 Global Cancer Statistics ranked CRC as the 2nd leading cause of cancer-related morbidity, with an estimated 881,000 cancer deaths worldwide and nearly 1.8 million newly diagnosed cases each year [1,5,6].

It has been widely established that the *KRAS* mutation pattern has a significant impact on the orientation of anticancer therapy. In this context, tumors harboring exon 2 *KRAS* mutations (codons 12 and 13) do not benefit from EGFR targeted therapies.

Interestingly, some wild-type *KRAS* exon 2 patients did not respond well to anti-EGFR therapy, proving that additional *RAS* mutations ( *KRAS* exons 3 and 4 or *NRAS* exons 2, 3, and 4) can negatively predict the success of anti-EGFR treatment.

In the present study, the frequencies of *KRAS* and *NRAS* gene mutations were determined in Tunisian patients with sporadic CRC. Additionally, we investigated correlations between these genetic mutations and clinicopathological features. Our results are consistent with previous studies in which 50% of colorectal cancers harbored a *RAS* mutation [29].

With a *KRAS* exon 2 mutation frequency of 41.7%, we were in accordance with Tunisian and worldwide studies in which frequencies ranged from 15% to 46%, respectively, [30–33] and from 30% to 50% (Table 4).

**Table 4:** KRAS mutation frequencies in different countries.

Country	KRAS mutation frequency	Reference
Australia	41.6%	[17]
China	47.2%	[21]
	42.56%	[49]
France	39.6%	[29]
Greece	41.3%	[22]
India	35.7%	[53]
Italy	50%	[52]
Romania	39.2%	[22]
USA	36.2%	[44]

Therefore, we noticed that *KRAS* mutations arise at similar frequencies in Tunisian patients as in other populations, and this may be attributed to the involvement of the same genes in sporadic colorectal carcinomas, regardless of the variation imposed by ethnicity, geographical distribution, dietary, lifestyle factors, and sensitivity to the different techniques used in previous studies.

In accordance with previous reports, 90% of *KRAS* mutations found in our cohort were located in codons 12 and 13. The majority occurred in codon 12 (85%) and (15%) in codon 13. The most frequently observed types of mutations were G>A transitions and G>T transversions [14,16,31,34–36].

We found that the most abundant mutations of codon 12 were G12D and G12V, while G13D is the predominant mutation in codon 13. These results are concordant with local Tunisian studies [30,31,33,37] and international ones [12,34,36,38–41].

Similar to the data in the literature, we also report a cluster of 4 mutation types (G12D, G12V, G12A, and G13D), which account for 84.8% (39/46) of *KRAS* exon 2 mutations [38,39,41].

Correlations between *KRAS* and *NRAS* mutational status and the different clinicopathological features are very controversial. Some previous reports pinpointed that the frequency of *KRAS* and *NRAS* mutation was associated with various clinicopathological criteria, but others did not.

Regarding *KRAS* exon 2, most of our results were consistent with the literature, notably the association with age, tumor location, and histology.

Our data showed that *KRAS* exon 2 mutations seemed to occur frequently in elderly patients. This result was supported by many other studies [20,41–43].

When it comes to tumor location, disparities have been reported where *KRAS* mutation rates were higher in the right-sided CRC tumors [2,36,40,44]. Our study, along with others, showed an association with the left side of the colon rather than the right [45–48].

The cause of the divergent findings between left- and right-side colon adenocarcinoma is still unclear [40]. It could be attributed to the complex origin and the exposure of left-sided luminal microenvironment to ingested carcinogens and mutagens [40].

Our data align with those reported in the literature that found that *KRAS* mutations showed a significant association with well-differentiated tumors but less with moderately differentiated tumors, with no or few *KRAS* mutations found in poorly differentiated tumors [2,29,31,35,36,46,48].

The association between *KRAS* mutations and mucinous histotype was reported in some studies [36,46,49] but denied in others [29,50,51], including ours.

Unlike *KRAS* mutations that are strongly implicated in colorectal cancer, *NRAS* alterations are rare and, to date, limited data on their mutation prevalence are available [36].

In our study, the *NRAS* mutation rate was 7.3%, similar to the only available Tunisian study, which reported 6.9% [37].

Our data shows that 12.5% of wild-type *KRAS* exon 2 patients carried a mutation in *NRAS* exons 2 and 3. Furthermore, recent data showed that 12%–17% of patients with wild-type *KRAS* exon 2 (codons 12/13) harbor a mutation in *KRAS* exons 3 and 4 and *NRAS* exons 2, 3, and 4 [25,52]. 

We observed that *NRAS* mutation incidence rates varied depending on the population, whereas Zhang reported a rate of 3.69% in a Chinese study [49], and 6% and 6.3% frequencies were described in Italian and Indian studies, respectively, [52,53] versus 9.57% in Greek and Romanian patients [22].

In our study, *NRAS* mutations were associated with early stages of cancer and the absence of lymph node metastasis.

Some studies have observed that *NRAS* mutations tended to occur in left-sided cancers and in women [54], while Russo et al. reported associations with rectal cancer and with patients 56 years or older [55].

Chang noted a correlation with the male sex [12]. Furthermore, Shen observed that these mutations were more frequent in distant metastasis tumors, and its rate varied with the different tumor stages [39]. Other studies did not find any correlations [29,36,37,49].

This divergence may be attributed to diverse ethnicities, genetic factors, geographical distributions, and diagnostic techniques. 

Nowadays, various techniques for assessing *RAS* mutation status are available, such as Sanger sequencing, high-resolution melt analysis, pyrosequencing, and next-generation sequencing techniques [56]. Though the tests vary in terms of sensitivity and specificity, no standard method has yet been endorsed for clinical practice [57].

In our study, we used the AmoyDx Mutation Detection Kit, a relatively simple real-time PCR assay that is fast and less prone to external contamination [58]. It is considered to be one of the most sensitive methods available in clinical molecular laboratories [59]. 

Due to its high sensitivity and accuracy, the AmoyDx *KRAS* real-time PCR kit has significantly higher mutation detection rates than Sanger DNA sequencing [58]. Therefore, AmoyDx real-time PCR is an effective and reliable tool for the clinical screening of somatic gene mutations in colorectal tumors [58].

However, we have to point out here that the present study was retrospective with a small sample size and focused only on *KRAS* exon 2 and *NRAS* exons 2, 3, and 4, preventing us from drawing any firm conclusions. Our database did not include any detailed information about adjuvant chemotherapy; hence, the patients’ adjuvant treatment was not analyzed in the current study.

## 5. Conclusion

In conclusion, we studied mutations of *KRAS* and *NRAS* genes in Tunisian CRC patients and their correlations with clinicopathological features. Our results show that in terms of incidence, *KRAS* and *NRAS* mutations occur at similar frequencies in Tunisian patients as in other populations. Meanwhile, clinicopathological features analysis showed both similarities and differences when contrasted to those reported in other studies. 

Consistent with the literature, *KRAS* exon 2 was associated with older patients, left-sided tumors, and greater differentiation. Otherwise, no association was found with other clinicopathological criteria such as sex, lymph node metastasis, tumor stage, or histological type.

In terms of *NRAS* mutations, our study showed an association with early stages of cancer and the absence of lymph node metastasis, different from various research studies that reported an association with other features like tumor location, sex, or age.

Therefore, screening for *KRAS* and *NRAS* mutation is crucial in guiding therapies and the selection of appropriate candidates, and in preventing unnecessary toxicity and costs for patients.

Given the importance of such molecular analysis, future studies can focus on the evaluation of other biomarkers suggested as having poor or no benefit from anti-EGFR therapy, such as *KRAS* exons 3 or 4, or *BRAF* .

## References

[ref1] (2018). GLOBOCAN estimates of incidence and mortality worldwide for 36 cancers in 185 countries. Global cancer statistics.

[ref2] (2018). Combined assessment of the TNM stage and BRAF mutational status at diagnosis in sporadic colorectal cancer patients. Oncotarget.

[ref3] (1994). Colorectal cancer incidence trend and projections in Tunisia (. Asian Pacific Journal of Cancer Prevention.

[ref4] (2011). Colorectal cancer in central Tunisia: increasing incidence trends over a 15-year period. Asian Pacific Journal of Cancer Prevention.

[ref5] (2013). Nutrition and colorectal cancer relationship in Tunisian population; beginning an answer. Immuno-analyse & Biologie Spécialisée.

[ref6] (2018). Malnutrition and risk factors in tunisian patients with colorectal cancer. Ibnosina Journal of Medicine and Biomedical Sciences.

[ref7] (1990). A genetic model for colorectal tumorigenesis. Cell.

[ref8] (2018). The molecular characteristics of colorectal cancer: implications for diagnosis and therapy (Review). Oncology Letters.

[ref9] (2017). -ras mutations as the earliest driving rorce in a subset of colorectal carcinomas. In Vivo.

[ref10] (1988). Genetic alterations during colorectal-tumor development. The New England Journal of Medicine.

[ref11] (2019). Impact of primary colorectal cancer location on the KRAS status and its prognostic value. BioMed Central Gastroenterology.

[ref12] (2016). Mutation spectra of RAS gene family in colorectal cancer. American Journal of Surgery.

[ref13] (2018). Genetic and epigenetic alterations of colorectal cancer. Intestinal Research.

[ref14] (2018). Mutations in KRAS codon 12 predict poor survival in Chinese patients with metastatic colorectal cancer. Oncology Letters.

[ref15] (2013). Kras gene mutation and RASSF1A, FHIT and MGMT gene promoter hypermethylation: indicators of tumor staging and metastasis in adenocarcinomatous sporadic colorectal cancer in Indian population. Public Library of Science ONE.

[ref16] (2017). The pattern of KRAS mutations in metastatic colorectal cancer: a retrospective audit from Sri Lanka. BioMed Central Research Notes.

[ref17] (2008). K-ras mutations and benefit from cetuximab in advanced colorectal cancer. The New England Journal of Medicine.

[ref18] (2009). American Society Of Clinical Oncology provisional clinical opinion: testing for KRAS gene mutations in patients with metastatic colorectal carcinoma to predict response to anti-epidermal growth factor receptor monoclonal antibody therapy. Journal of Clinical Oncology: Official Journal of the American Society of Clinical Oncology.

[ref19] (2009). NCCN Clinical practice guidelines in oncology: colon cancer. Journal of the National Comprehensive Cancer Network.

[ref20] (2013). Detection of low-abundance KRAS mutations in colorectal cancer using microfluidic capillary electrophoresis-based restriction fragment length polymorphism method with optimized assay conditions. Public Library of Science One.

[ref21] (2018). Comprehensive analysis of the relationship between RAS and RAF mutations and MSI status of colorectal cancer in northeastern China. Cellular Physiology and Biochemistry: International Journal of Experimental Cellular Physiology, Biochemistry, and Pharmacology.

[ref22] (2014). KRAS, NRAS and BRAF mutations in Greek and Romanian patients with colorectal cancer: a cohort study. British Medical Journal Open.

[ref23] (2017). Frequency of RAS mutations (KRAS, NRAS, HRAS) in human solid cancer. Eurasian Journal of Medicine and Oncology.

[ref24] (2010). Effects of KRAS, BRAF, NRAS, and PIK3CA mutations on the efficacy of cetuximab plus chemotherapy in chemotherapy-refractory metastatic colorectal cancer: a retrospective consortium analysis. The Lancet Oncology.

[ref25] (2013). Panitumumab-FOLFOX4 treatment and RAS mutations in colorectal cancer. The New England Journal of Medicine.

[ref26] (2016). Extended RAS gene mutation resting in metastatic colorectal carcinoma to predict response to anti-epidermal growth gactor receptor monoclonal antibody therapy:. American Society of Clinical Oncology Provisional Clinical Opinion Update 2015 Summary. Journal of Oncology Practice.

[ref27] (2010). WHO Classification of Tumours of the Digestive System.

[ref28] (2017). AJCC Cancer Staging Manual.

[ref29] (2018). Association between clinicopathological characteristics and RAS mutation in colorectal cancer. Modern Pathology: An Official Journal of the United States and Canadian Academy of Pathology.

[ref30] (2015). Mutations du gène KRAS chez les patients du sud tunisien atteint de cancer colorectal: signification clinique. Journal de l’Information Médicale de Sfax.

[ref31] (2013). KRAS mutations in colorectal cancer from Tunisia: relationships with clinicopathologic variables and data on TP53 mutations and microsatellite instability. Molecular Biology Reports.

[ref32] (2011). KRAS mutation detection in Tunisian sporadic coloractal cancer patients with direct sequencing, high resolution melting and denaturating high performance liquid chromatography. Cancer Biomarkers.

[ref33] (2013). The prevalence and prognostic significance of KRAS mutation in bladder cancer, chronic myeloid leukemia and colorectal cancer. Molecular Biology Reports.

[ref34] (2009). Frequency and type of KRAS mutations in routine diagnostic analysis of metastatic colorectal cancer. Pathology, Research and Practice.

[ref35] (2012). Mutation pattern of KRAS and BRAF oncogenes in colorectal cancer patients. Neoplasma.

[ref36] (2015). Molecular spectrum of KRAS, NRAS, BRAF and PIK3CA mutations in Chinese colorectal cancer patients: analysis of 1,110 cases. Scientific Reports.

[ref37] (2019). KRAS and NRAS pyrosequencing screening in Tunisian colorectal cancer patients in 2015. Heliyon.

[ref38] (2013). Clinical utility of KRAS and BRAF mutations in a cohort of patients with colorectal neoplasms submitted for microsatellite instability testing. Clinical Colorectal Cancer.

[ref39] (2013). Effectors of epidermal growth gactor receptor pathway: the genetic profiling of KRAS, BRAF, PIK3CA, NRAS mutations in colorectal cancer characteristics and personalized medicine. Public Library of Science One.

[ref40] (2014). Characterization of rare transforming KRAS mutations in sporadic colorectal cancer. Cancer Biology & Therapy.

[ref41] (2013). Direct sequencing is a reliable assay with good clinical applicability for KRAS mutation testing in colorectal cancer. Cancer Biomarkers.

[ref42] (2018). Mutation status and prognostic values of KRAS, NRAS, BRAF and PIK3CA in 353 Chinese colorectal cancer patients. Scientific Reports.

[ref43] (2015). KRAS and BRAF gene mutations and DNA mismatch repair status in Chinese colorectal carcinoma patients. World Journal of Gastroenterology.

[ref44] (2014). Analyses of clinicopathological, molecular, and prognostic associations of KRAS codon 61 and codon 146 mutations in colorectal cancer: cohort study and literature review. Molecular Cancer.

[ref45] (2018). Difference between left-sided and right-sided colorectal cancer: a focused review of literature. Gastroenterology Research.

[ref46] (2009). Frequency and clinicopathological associations of K-ras mutations in Venezuelan patients with colo-rectal cancer. Investigacion Clinica.

[ref47] (2007). K-ras mutation detection in colorectal cancer using the pyrosequencing technique. Pathology - Research and Practice.

[ref48] (2014). Prevalence of KRAS mutations in metastatic colorectal cancer: a retrospective observational study from India. Indian Journal of Cancer.

[ref49] (2018). Deficient mismatch repair and RAS mutation in colorectal carcinoma patients: a retrospective study in Eastern China. Peer The Journal of Life and Environmental Sciences.

[ref50] (2005). Mucinous carcinomas of the colorectum have distinct molecular genetic characteristics. International Journal of Oncology.

[ref51] (2006). BRAF mutation, CpG island methylator phenotype and microsatellite instability occur more frequently and concordantly in mucinous than non-mucinous colorectal cancer. International Journal of Cancer.

[ref52] (2015). Role of NRAS mutations as prognostic and predictive markers in metastatic colorectal cancer. International Journal of Cancer.

[ref53] (2017). Prevalence and coexistence of KRAS, BRAF, PIK3CA, NRAS, TP53, and APC mutations in Indian colorectal cancer patients: next-generation sequencing–based cohort study. Tumor Biology.

[ref54] (2010). NRAS mutations are rare in colorectal cancer. Diagnostic Molecular Pathology: The American Journal of Surgical Pathology.

[ref55] (2014). Mutational analysis and clinical correlation of metastatic colorectal cancer. Cancer.

[ref56] (2016). KRAS mutation testing in colorectal cancer: the model for molecular pathology testing in the future. Colorectal Cancer.

[ref57] (2016). A comparison of four methods for detecting KRAS mutations in formalin-fixed specimens from metastatic colorectal cancer patients. Oncology Letters.

[ref58] (2012). Comparative screening of K-ras mutations in colorectal cancer and lung cancer patients ssing a novel real-time PCR with ADx-K-ras kit and sanger DNA sequencing. Cell Biochemistry and Biophysics.

[ref59] (2018). Development of ultra-short PCR assay to reveal BRAF V600 mutation status in Thai colorectal cancer tissues. Public Library of Science ONE.

